# Effects of groundwater depth on ecological stoichiometric characteristics of assimilated branches and soil of two desert plants

**DOI:** 10.3389/fpls.2023.1225907

**Published:** 2023-08-08

**Authors:** Xue Wu, Xueying Wang, Pengqi Wang, Yuanting Gu, Yan Li

**Affiliations:** ^1^ College of Ecology and Environment, Xinjiang University, Urumqi, China; ^2^ Key Laboratory of Oasis Ecology, Ministry of Education, Urumqi, China; ^3^ Ecological Postdoctoral Research Station, Xinjiang University, Urumqi, China; ^4^ Xinjiang Jinghe Observation and Research Station of Temperate Desert Ecosystem, Ministry of Education, Jinghe, China; ^5^ Xinjiang Institute of Ecology and Geography, Chinese Academy of Sciences, Urumqi, China; ^6^ Fukang Station of Desert Ecology, Xinjiang Institute of Ecology and Geography, Chinese Academy of Sciences, Urumqi, China

**Keywords:** arid region, desert plants, groundwater depth, *haloxylon*, ecological stoichiometry

## Abstract

Groundwater plays a crucial role in regulating plant growth in arid regions and has significant effects on plant physiological mechanisms. However, research on the influence of groundwater change on plant ecological stoichiometry is still limited. Therefore, this study was carried out to obtain the variations in assimilated branches and soil ecological stoichiometry of two dominant species in the Gurbantunggut Desert (*Haloxylon ammodendron* and *Haloxylon persicum*) at different groundwater depths to reveal the responses of desert plants to groundwater depth changes. The results showed that (1) *H. persicum* branches’ stress tolerance indicators (C:N, C:P) are higher, while nutritional indicators (N:P) are lower. The soil nutrient of *H. ammodendron* is richer. (2) The ecological stoichiometry varied significantly along the groundwater gradient. With the deepening of groundwater, the branches C, N and P increased, and the variation in element ratio was inconsistent. Most of the soil properties was inversely proportional to the depth of groundwater. (3) Groundwater depth was a vital environmental factor affecting the assimilated branches ecological stoichiometry. Soil properties also had a significant influence on element accumulation in assimilated branches. (4) Regulating the allocation of branches ecological stoichiometry is an adaptation of two *Haloxylon* species to cope with local hydrological conditions changes. These findings provide novel insights into desert plant responses to different groundwater conditions within fragile desert ecosystems and may have implications for the implementation of effective measures related to the stability and sustainability of desert ecosystems.

## 1 Introduction

Ecological stoichiometry is a new effective way of exploring soil−plant interactions and the characteristics of ecosystem element cycling. C, N and P are the most basic constituent elements of plants and soil nutrients (Fan et al., 2015). The investigation of assimilated branches and soil nutrients can illuminate the interdependent relationships among soil nutrients and is of great significance in regulating the cycling of soil elements (Gao et al., 2019). In recent years, research on soil and plant ecological stoichiometry has developed rapidly. The ecological stoichiometry characteristics of different plant organs, different ecological scales (populations, communities, ecosystems), the effects of human activities on them and their application value have been investigated profoundly (Zeng et al., 2016; Tian et al., 2021; Jiang et al., 2022; Li et al., 2022; Sun et al., 2022; Liu et al., 2023). Existing studies have revealed the material cycle process at different scales and the operating mechanism of different ecosystems, which has profound significance for understanding and restoring ecosystems. However, the relevant studies were more focused on forest and grassland ecosystems. In arid zones, studies on ecological stoichiometry have mainly focused on their quantitative and spatial variabilities and the correlation between plant ecological stoichiometry and climatic factors (Xu and Li, 2006; Tang et al., 2018; He et al., 2019; Luo et al., 2022; Nan et al., 2022). However, most studies focused on precipitation, while few have considered groundwater depth. Currently, significant changes in climate and hydrological conditions are occurring, causing important effects on soil−plant interactions in the arid environment of desert ecosystems (Sardans and Penuelas, 2012). Ecological stoichiometry can be an effective way to study the nutrient absorption and utilization of desert plants and the response mechanisms of desert ecosystems to environmental changes.

Groundwater is a stable source that can be continuously provided for plant and soil organisms as long as the plant roots or soil capillaries can access the vicinity of it (Fan, 2015; Mendes et al., 2016). Different depths of groundwater have significant effects on vegetation density, plant growth (Martinetti et al., 2021), and soil properties (Chen et al., 2009). The physiological metabolism of individuals can also be significantly affected (Niklas and Cobb, 2005; Barbeta et al., 2014). In recent decades, along with urban expansion and the development of irrigated agriculture, groundwater availability and stability have decreased dramatically (Klöve et al., 2011). Moreover, global climate change is causing increased evaporation (Gall et al., 1992), making surface water more vulnerable to evaporation and loss. The scarcity of precipitation in arid zones makes groundwater the most crucial ecological factor for plant survival because it is an important water source that desert plants can rely on and plays a critical role in maintaining desert ecosystem function (Wu et al., 2019a). In this region, overexploitation of soil and water resources has reduced the sustainability of groundwater resources, causing a major constraint on the ecological security of local oasis farmlands (Wu et al., 2019a; Ling et al., 2020). Therefore, it is important to explore the effects of groundwater change on local soil−plant systems.

The Gurbantunggut Desert, located in the middle of the Junggar Basin, is the largest fixed and semifixed desert in China. The southern part is influenced by oasis and is richer in vegetation than deserts of the same latitude; thus, it has become an important repository of heat-, salt- and drought-tolerant species in China (Zhang et al., 2008). *Haloxylon ammodendron* and *Haloxylon persicum* are plants of the *Haloxylon* genus. After long-term natural selection, they have shown strong adaptability to habitats, so they are important for wind control and sand control in the Gurbantunggut Desert (Yue et al., 2020). Extensive research has been conducted on *H. ammodendron* and *H. persicum*, mainly focusing on the effects of environmental change (such as topographic change, precipitation change, and nitrogen addition) on their community structure, growth, development, physiological performance, and water use pattern (Xu and Li, 2006; Hao and Li, 2022; Song et al., 2010; Wu et al., 2019b; He et al., 2022; Qiang et al., 2022). However, little research has been conducted on the links between hydrological conditions and their ecological stoichiometry.

Therefore, this study analyzed the assimilated branches and soil ecological stoichiometry characteristics of two *Haloxylon* species along groundwater gradients in the Gurbantunggut Desert and explored their relationship. The aim is to explore the response in ecological stoichiometry of desert plants to groundwater changes and their correlation with soil environmental factors, revealing their physiological and ecological survival mechanisms, to provide a theoretical basis for the protection and restoration of desert ecosystems under changes in local hydrological conditions in the future. We hypothesize that as groundwater depth changes, *Haloxylon* species ecological stoichiometry exhibit adapted changes, although due to the homeostasis mechanism and adaptive adjustment, this change may not be linear or even weak.

## 2 Materials and methods

### 2.1 Study area and plant species

**Figure 1:**
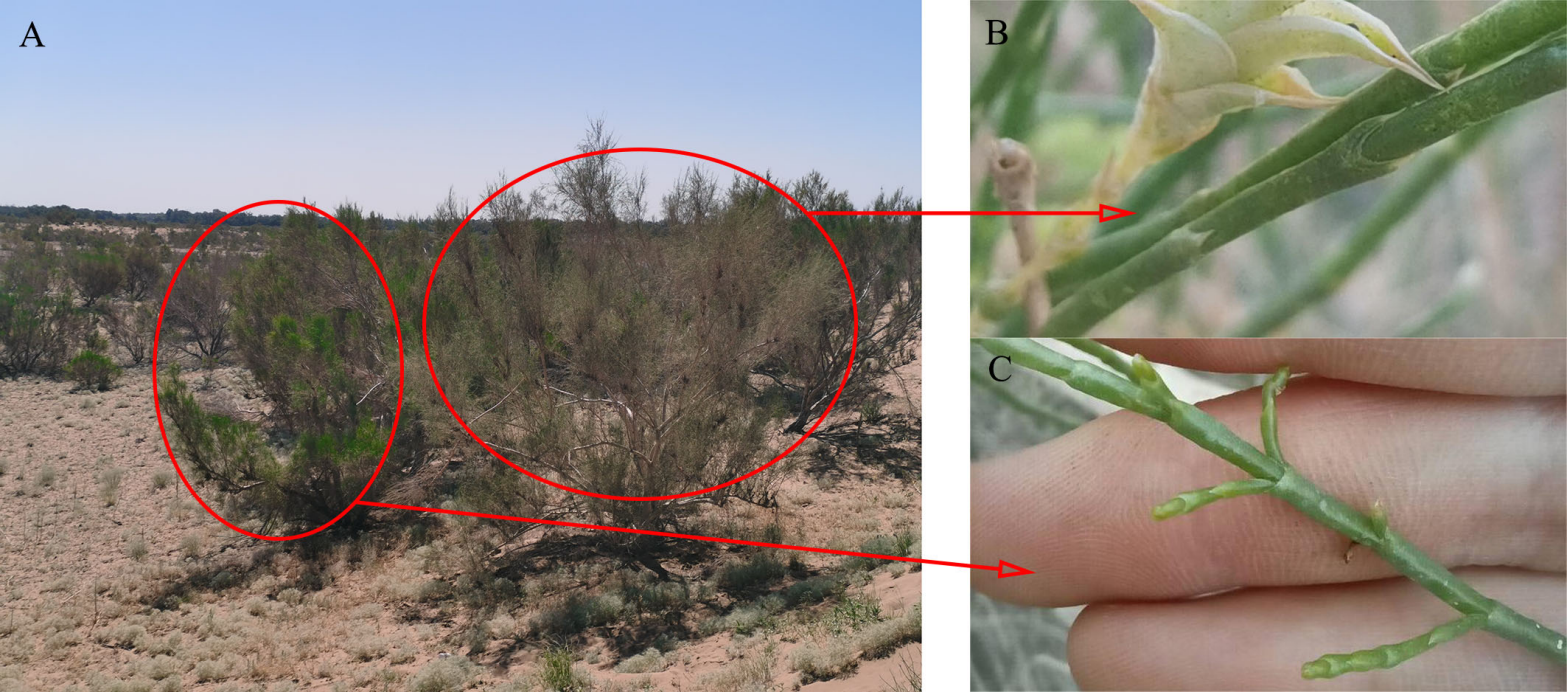
The individual form and assimilated branches of *H. persicum* **(A, B)** and *H. ammodendron* **(A, C)**.

The study was conducted in the southeastern Gurbantunggut Desert near the Fukang Desert Ecosystem Observation and Experiment Station, Chinese Academy of Sciences. The region has a typical temperate continental climate, with dry and hot summers and quite cold and snowy winters. The temperatures vary considerably during the year, ranging from a minimum of -42.2°C to a maximum of 44.2°C. The annual average wind speed is 3.2 m/s, with prevailing northwesterly winds (Zhang et al., 2007). The average annual evaporation is approximately 1000 mm, while the average annual precipitation is only 70-150 mm, part of which occurs in the form of snowfall, accounting for approximately 18.7% of the total annual precipitation (Zhou et al., 2012). Rapid snow melting in the early spring (March to April) initiates a brief bloom of ephemeral germination, but most of the time, only sparse shrubs and small trees grow on the surface (such as *H. ammodendron* and *H. persicum*), with less than 10% vegetation cover (Zhou et al., 2012). Sandy soils are the main soil type in the study area, with small particles, which flow easily in the wind and have good permeability and are thus susceptible to nutrient loss and become infertile (Zhang and Chen, 2002). Water from oasis recharges groundwater from its outer desert, so that the groundwater is buried at a depth of only about 3-4 m on the southern edge of the Gurbantunggut Desert, but more than tens of meters in its hinterland (Chen, 2017). Moreover, seasonal dynamics in groundwater existed. According to the observation of the wells, groundwater rises in the period of snow-melting. After that, the groundwater level decreased and remain stable, without significant change. *H. ammodendron* and *H. persicum* are constructive species in the Gurbantunggut Desert (Wu et al., 2019a), both belonging to the *Chenopodiaceae*. The two species, with similar canopy and assimilated branches morphology, have developed adaptive features in structure and physiology, which are mainly manifested in the degeneration of the assimilated branches into green assimilated branches to carry out photosynthesis and have higher efficiency (Li and Li, 1981; Guo et al., 2004) (**Figure 1**). However, the dominant topography of their distribution is significantly different. *H. persicum* is distributed at the top of sandy dunes with lower soil nutrient and salt contents due to its high nutrient conservation rate (Qian et al., 2008; Yue et al., 2020). *H. ammodendron* occupies interdune depressions, where soil nutrient and salt contents are higher because of high nutrient uptake efficiency (Yue et al., 2020; Li et al., 2023).

### 2.2 Plot setting and groundwater depth measurement

**Figure 2:**
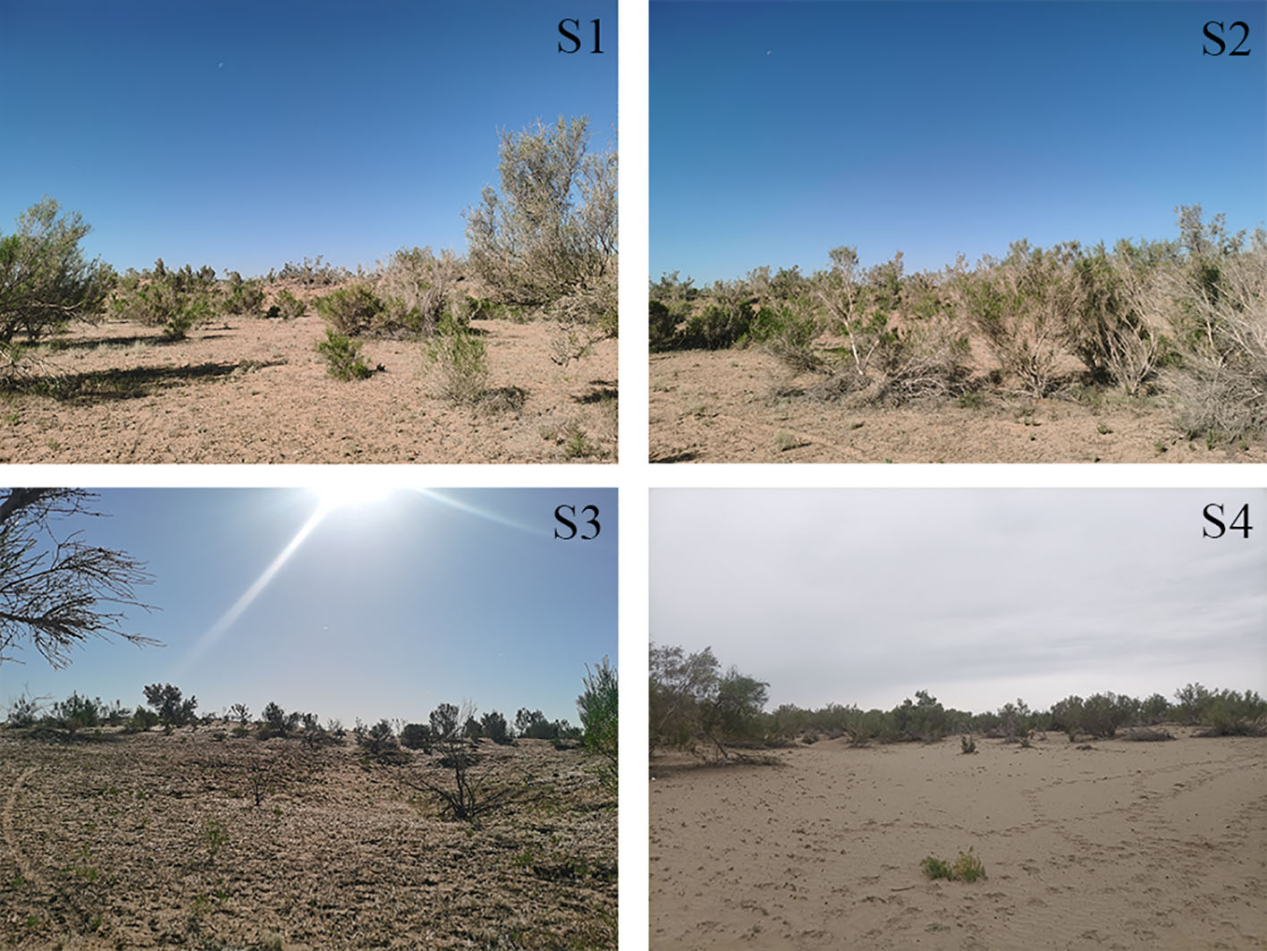
Schematic diagram of sampling site landscape. S1-S4 correspond to site 1-4.

From the southern edge of the Gurbantunggut Desert to the hinterland, the depth of groundwater gradually increases, resulting in a natural groundwater gradient. In October 2015 and April 2017, 25 groundwater observation wells were drilled in the southern Gurbantunggut Desert, with groundwater depth variation range of 4-16m, and four areas with groundwater depth of 4m, 7m, 13m, and 16m were selected for experimentation (**Figure 2**). The dunes have an average height of 11 meters (Dai et al., 2014), so for *H. persicum*, the depth of groundwater is 15m, 18m, 24m, and 27m. Based on information on the groundwater gradient and the changes in plant communities, four typical study sample sites were finally identified. The coordinate locations and altitude are shown in **Table 1**.

**Table 1:** The coordinate locations of the sampling points and the vegetation density.

Site	Groundwater depth (m)	Longitude	Latitude	Interdune altitude (m)	Dune-tops altitude (m)	Vegetation density (No./ha)
1	4	87°55′02″E	44°22′56″N	437.4	448.4	2296.67 ± 193.76
2	7	87°55′22″E	44°24′32″N	441.1	452.1	1523.33 ± 135.32
3	13	87°54′30″E	44°25′38″N	438.2	449.2	1170.00 ± 25.17
4	16	87°53′49″E	44°30′35″N	440.6	451.6	206.67 ± 44.85

### 2.3 Experimental design and measurements

The experiments were conducted in June-July 2017, when the plants were in full growth. Three 100 m*10 m plots were set at the depth of each groundwater depth. *H. ammodendron* and *H. persicum* in the plots were counted, and the actual number of individuals was converted to vegetation density, in No/ha (**Table 1**). At each sample point, five *H. ammodendron* plants and five *H. persicum* plants with similar morphology and health status were selected. Their healthy, pest-free assimilated branches were collected from different directions, mixed and stored in kraft envelopes at low temperature, for a total of 40 plant assimilated branches samples.

Along with the plant sampling, soils were sampled from different layers under the canopy of the two species. After stripping off the litter, soil samples were collected with a 5 cm diameter soil auger at depths of 20, 40, 60, 80, 100, 140, 180, 220, 260 and 300 cm. Four replicate groups were taken for each layer. In total, 320 soil samples were collected. Each soil sample was divided into two parts, one in a Ziplock bag for the determination of soil physical and chemical properties and the other in an aluminum box for the determination of soil water content.

### 2.4 Measurement of assimilated branches ecological stoichiometry

The measured assimilated branches indicators included assimilated branches organic carbon (LOC), total nitrogen (LTN) and total phosphorus (LTP) and their stoichiometric ratios. Before the measurement, assimilated branches samples were dried to constant weight in an oven at 70°C, ground with a grinder (NM200, Retsch, Haan, Germany), passed through a 0.15 mm sample sieve and stored in sealed bags. Assimilated branches organic carbon was determined by the potassium dichromate-sulfuric acid oxidation method. Assimilated branches total nitrogen was determined using a fully automatic Kjeldahl analyzer (The Swedish FOSS, Kjeltec™8400). The total phosphorus of the assimilated branches was determined using a molybdenum antimony anti-colorimetric method, and their C/N, C/P and N/P were calculated using elemental values.

### 2.5 Determination of soil ecological stoichiometry and other properties

The measured soil characteristics included major chemical and physical properties, including soil organic carbon (SOC), total nitrogen (STN), total phosphorus (STP) content and their stoichiometric ratios, pH, water content, and electronic conductivity (EC).

Soil samples that had been air-dried for two weeks were subsequently ground to a fine powder and passed through a 0.25 mm sieve before being stored in sealed bags. Soil organic carbon was determined by the potassium dichromate-sulfuric acid oxidation method. Total nitrogen was determined using a fully automatic Kjeldahl analyzer (The Swedish FOSS, Kjeltec™8400). Total phosphorus was determined using the sulfuric acid-perchlorate-boiled-molybdenum antimony colorimetric method. Their C/N, C/P and N/P were calculated using elemental values. The soil water content (SWC) was determined using the drying method. Soil pH and electrical conductivity (EC) were measured using the electrode potential method in the supernatant of 1:5 soil−water mixtures.

### 2.6 Statistical analyses

The Shapiro-Wilk test is used for normality testing. Levene’s test was used to test for the homogeneity of variance between groundwater. For the differences in soils and plants (**Tables 2**, **3**), if the variance is normally distributed and the variance is homogeneous, the t-test is used, otherwise the Mann-Whitney U test is used. Two-way ANOVA was used to determine the effects of groundwater depth, soil depth, and their interaction on soil properties. When the variance was equal, comparison of means using Duncan’s multiple comparisons was performed. When the variance is unequal, comparison of means is performed using T2 Tamhane’s test (α = 0.05). Differences between species were tested for assimilated branches and soil ecological chemometrics using t tests (α = 0.05). The linear regression method was used to determine the relationship between assimilated branches or soil characteristics and the depth of groundwater (soil samples from the same site at different depths were averaged and plotted as the sample points). The relationship between assimilated branches indicators and soil characteristics for *H. ammodendron* and *H. persicum* was determined using redundancy analysis (RDA). Data were collated and processed using Microsoft Excel 2019. Analysis and graphing were performed using Origin 2022, Canoco 5, and R 4.2.3.

**Table 2:** Leaf ecological stoichiometry characteristics.

	*Haloxylon persicum*		*Haloxylon ammodendron*	
	Mean ± SE	CV (%)	Mean ± SE	CV (%)
LOC (g/kg)	392.36 ± 40.23 A	10.73	310.01 ± 43.83 B	14.37
LTN (g/kg)	7.19 ± 1.06 B	16.66	8.29 ± 0.74 A	11.1
LTP (g/kg)	0.88 ± 0.10	12.6	0.83 ± 0.09	13.85
C:N	54.60 ± 9.35 A	18.59	38.68 ± 6.52 B	18.70
C:P	458.54 ± 82.54 A	18.34	377.49 ± 36.41 B	11.84
N:P	8.22 ± 0.45 B	9.43	10.06 ± 1.06 A	12.38

CV, Coefficient of variation. The different capital letters in the same row represent significant interspecies differences (P < 0.05).

**Table 3:** Soil physical and chemical properties.

	*Haloxylon persicum*		*Haloxylon ammodendron*	
	Mean ± SE	CV (%)	Mean ± SE	CV (%)
SOC (g/kg)	0.50 ± 0.08 B	37.49	0.63 ± 0.08 A	47.09
STN (g/kg)	0.04 ± 0.01 B	46.09	0.07 ± 0.01 A	50.88
STP (g/kg)	0.17 ± 0.01 B	10.33	0.34 ± 0.04 A	20.95
pH	9.00 ± 0.22 B	3.64	9.22 ± 0.14 A	3.68
SWC (%)	0.93 ± 0.18 B	33.04	2.97 ± 1.80 A	117.41
EC	13.48 ± 2.72 B	70.83	78.66 ± 15.06 A	53.84
C:N	17.24 ± 2.60 A	25.84	10.12 ± 0.94 B	28.15
C:P	2.59 ± 1.90 A	45.84	1.89 ± 0.11 B	38.35
N:P	0.25 ± 0.03	49.87	0.22 ± 0.02	42.17

CV, Coefficient of variation. The different capital letters in the same row represent significant differences (P < 0.05).

## 3 Results

### 3.1 Statistical analysis of assimilated branches and soil ecological chemometric characteristics

According to **Table 2**, significant differences were observed between the assimilated branches ecological stoichiometry and soil characteristics of the two plants (*P* < 0.05), with the exception of LTP and soil N:P. Notably, *H. persicum* exhibited significantly higher LOC, assimilated branches C:N and C:P values than *H. ammodendron*, while LTN and N:P were significantly lower. Furthermore, the mean assimilated branches N:P for both were less than 11, emphasizing their potential nitrogen limitation.

In terms of soil characteristics, *H. ammodendron* exhibited significantly higher values of SOC, STN, STP, SWC, pH and EC than *H. persicum* (P < 0.05). The stoichiometric ratios (C:N, C:P, N:P) were smaller in *H. ammodendron*, with significant differences observed only in C:N and C:P (P < 0.05). Soil pH was alkaline for both species, with values exceeding 9 and showing minimal variation (CV < 4%).

**Table 2** shows that the variation in assimilated branches stoichiometry was comparatively small, with coefficients of variation ranging between 9% and 20% and mostly exhibiting minor differences between the two plant species. In contrast, other soil properties displayed moderate to strong levels of variability, as presented in **Table 3**. Specifically, the variability of SOC, STN, and STP in soils under the canopy of *H. ammodendron* exceeded that of *H. persicum*. Meanwhile, SWC in soils under the *H. ammodendron* canopy exhibited the highest variation, with a coefficient of variation exceeding 115%.

### 3.2 Assimilated branches ecological stoichiometry at different groundwater depths

**Figure 3:**
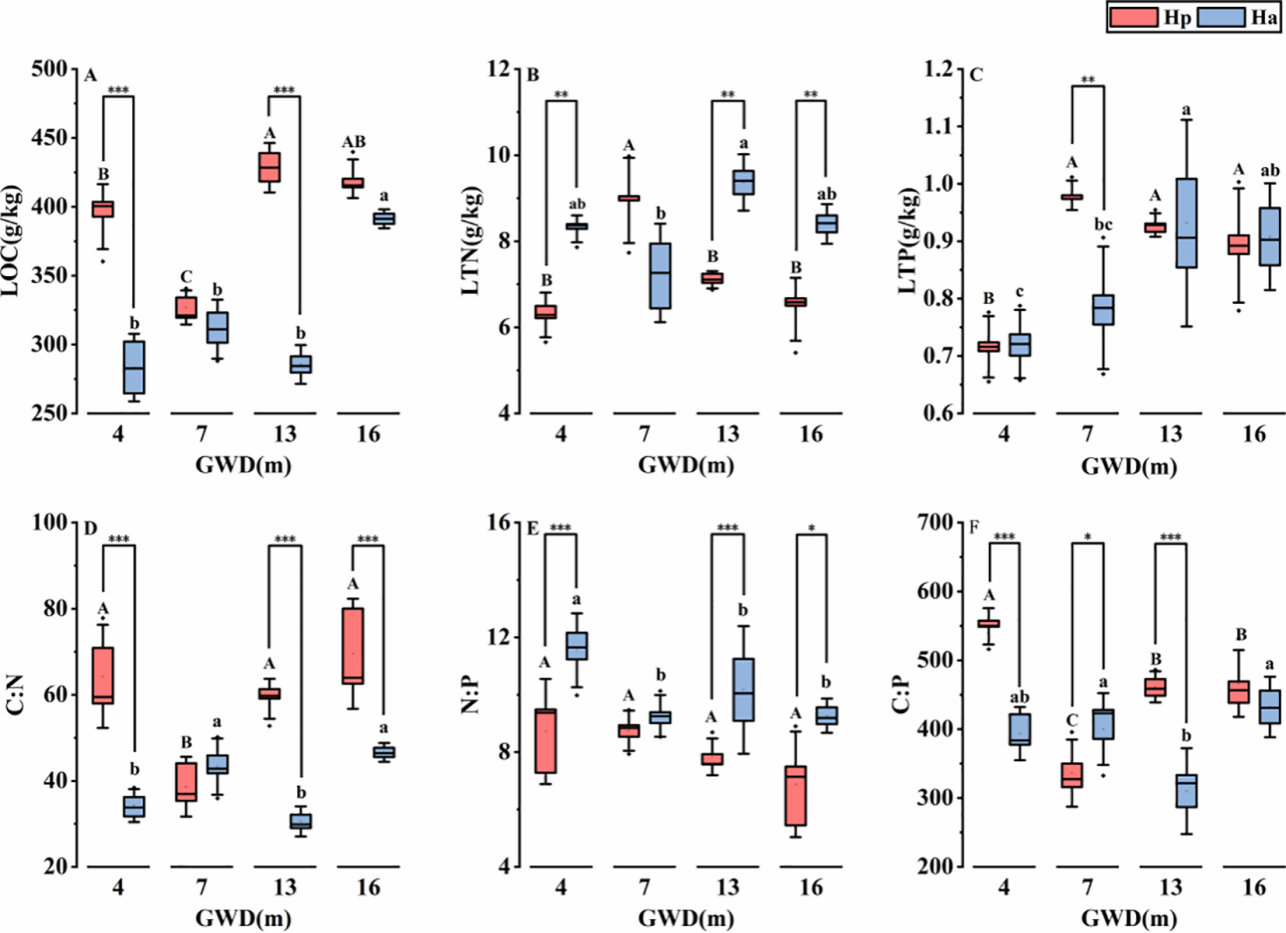
Ecological stoichiometry of *H. persicum* and *H. ammodendron* assimilated branches at different depth of groundwater. GWD: Groundwater depth; *P* < 0.001***, *P* < 0.01**, *P* < 0.05*. Different letters indicate significant differences in the assimilated branches ecological stoichiometry of *H. persicum* (lowercase) or *H. ammodendron* (Ha) respectively. LOC, assimilated branches organic carbon **(A)**; LTN, assimilated branches total nitrogen **(B)**; LTP, assimilated branches total phosphorus **(C)**; C:N, assimilated branches carbon-nitrogen ratio **(D)**; N:P, assimilated branches nitrogen-phosphorus ratio **(E)**; C:P, assimilated branches carbon-phosphorus ratio **(F)**.

The results presented in **Figure 3** demonstrate significant variations in the assimilated branches ecological stoichiometric characteristics of both plant species across different groundwater depths (*P* < 0.05), with the exception of *H. persicum* N/P ratios. In particular, the assimilated branches LOC of *H. ammodendron* increased significantly at deeper groundwater depths. The highest LTN and LTP values were observed at 13 m depth of groundwater, concomitant with a significant decrease in assimilated branches C:N and C:P values. Conversely, at 7 m depth of groundwater, *H. persicum* exhibited the lowest LOC and the highest LTN and LTP values, displaying significantly lower assimilated branches C:N and C:P ratios compared to other depths. It is speculated that groundwater depths of 7 m and 13 m may represent pivotal points for understanding the ecological stoichiometry of *H. ammodendron* and *H. persicum*, respectively.

Significant interspecific differences were also observed in the assimilated branches ecological stoichiometry of the two plant species. Specifically, compared to *H. ammodendron*, *H. persicum* exhibited greater LOC at all groundwater depths, while LTN was significantly lower at the 4 m, 13 m, and 16 m groundwater gradients (*P* < 0.05). Additionally, the assimilated branches N:P values of *H. persicum* were less than those of *H. ammodendron*. Assimilated branches C:N and C:P ratios were greater in *H. persicum* than in *H. ammodendron*, except at the 7 m groundwater depth.

### 3.3 Soil ecological stoichiometry at various groundwater depths

By two-way ANOVA, **Table 4** indicates significant differences in soil ecological stoichiometry with varying groundwater depths and soil depths, except for soil C:N ratios. The results indicated that SOC in *H. persicum* soils, as well as soil N:P in *H. ammodendron* soils, were both significantly influenced by groundwater depth and soil depth (*P* < 0.05), but with a weak interaction effect between the two (*P* > 0.05).

**Table 4:** Effects of groundwater depth, soil depth and their interaction on soil ecological chemometric characteristics.

		*Haloxylon persicum*			*Haloxylon ammodendron*		
		GWD	SD	GWD*SD	GWD	SD	GWD*SD
SOC (g/kg)	*F*	13.196	9.482	1.45	15.57	38.66	2.27
	*P*	**<0.001**	**<0.001**	0.090	**<0.001**	**<0.001**	**0.001**
STN (g/kg)	*F*	6.478	17.044	2.747	24.472	33.897	0.843
	*P*	**<0.001**	**<0.001**	**<0.001**	**<0.001**	**<0.001**	**<0.001**
STP (g/kg)	*F*	11.512	2.187	2.388	28.371	13.548	2.792
	*P*	**<0.001**	**0.0274**	**<0.001**	**<0.001**	**<0.001**	**<0.001**
C:N	*F*	1.302	0.846	0.585	3.206	0.924	1.128
	*P*	0.277	0.575	0.946	**0.026**	0.507	0.320
C:P	*F*	11.969	9.047	1.725	2.977	20.827	1.687
	*P*	**<0.001**	**<0.001**	**0.0246**	**<0.001**	**<0.001**	**0.0297**
N:P	*F*	7.519	16.843	2.962	9.169	25.277	1.272
	*P*	**<0.001**	**<0.001**	**<0.001**	**<0.001**	**<0.001**	0.190

GWD, Groundwater depth; SD, Soil depth. The symbol "*" indicates the interaction of GWD and SD. Bold values indicate P<0.001.

The C:N ratios in *H. ammodendron* soils were found to be influenced by groundwater depth (*P* < 0.05) but were not significantly affected by soil depth or their interaction (*P* > 0.05). In contrast, the GWD, SD, and their interaction had no significant impact on the C:N ratios of the soil of *H. persicum* (*P* > 0.05).

**Figure 4:**
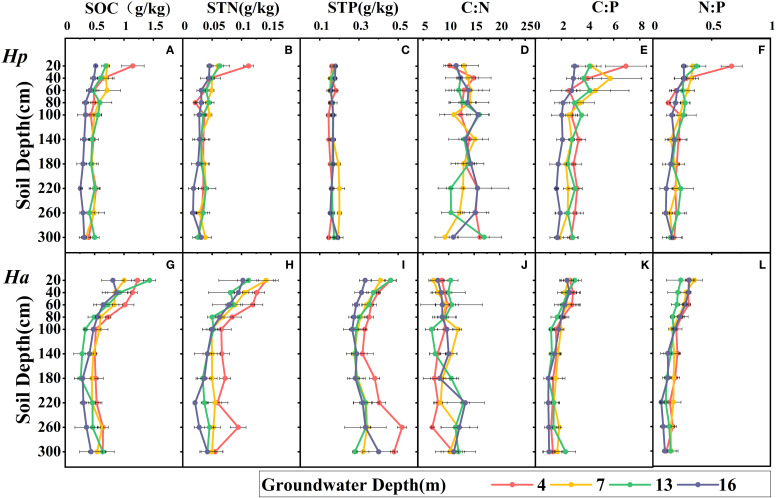
Variation in soil ecological stoichiometry with groundwater depth and soil depth under crowns. Ha, *H. ammodendron*; Hp, *H. persicum*. The four lines represent the changes under different GWDs. The black line segment is the error bar. The A B C D E F are *H. persicum*, and G H I J K L are *H. ammodendron*. SOC, soil organic carbon **(A, G)**; STN, soil total nitrogen **(B, H)**; STP, soil total phosphorus **(C, I)**; C:N, soil carbon-nitrogen ratio **(D, J)**; C:P, soil carbon-phosphorus ratio **(E, K)**; N:P, soil nitrogen-phosphorus ratio **(F, L)**.

Among various groundwater and soil depths, the soil ecological stoichiometry of both species was found to be significantly heterogeneous (**Table 4** and **Figure 4**). The levels of SOC, STN, soil C:P and soil N:P decreased with increasing soil depth, which was more pronounced in shallow soils than in deeper soils. The variation in C:N with groundwater depth and soil depth was not uniform, indicating that the influencing factors may be intricate. In *H. persicum*, SOC, STN, soil C:P and soil N:P decreased with increasing groundwater depth in shallow soils, while STP increased significantly at 7 m groundwater depth in the deeper layers, which may be related to the higher content of LTP in *H. persicum* assimilated branches at that depth. On the other hand, SOC variation in *H. ammodendron* soil was the greatest at 13 m groundwater depth, and STP content slightly increased at the deeper layer, being significantly higher at 4 m depth than at other depths. The differences in soil C:N at each depth were not significant in the shallow layers but diverged with increasing depth of the soil layer. The variation in soil C:N mainly indicated an increase with increasing groundwater depth. The two species also exhibited differences in soil ecological stoichiometry. Significantly, *H. ammodendron* had higher concentrations of SOC, STN, and STP than *H. persicum*. Additionally, the stoichiometric ratios of *H. persicum* were more extensively varied than others. *H. ammodendron* had significantly higher levels of SOC, STN, and STP in shallow soils and more pronounced changes with increasing soil depth.

### 3.4 Relationship between assimilated branches or soil ecological stoichiometry and groundwater depth

**Figure 5:**
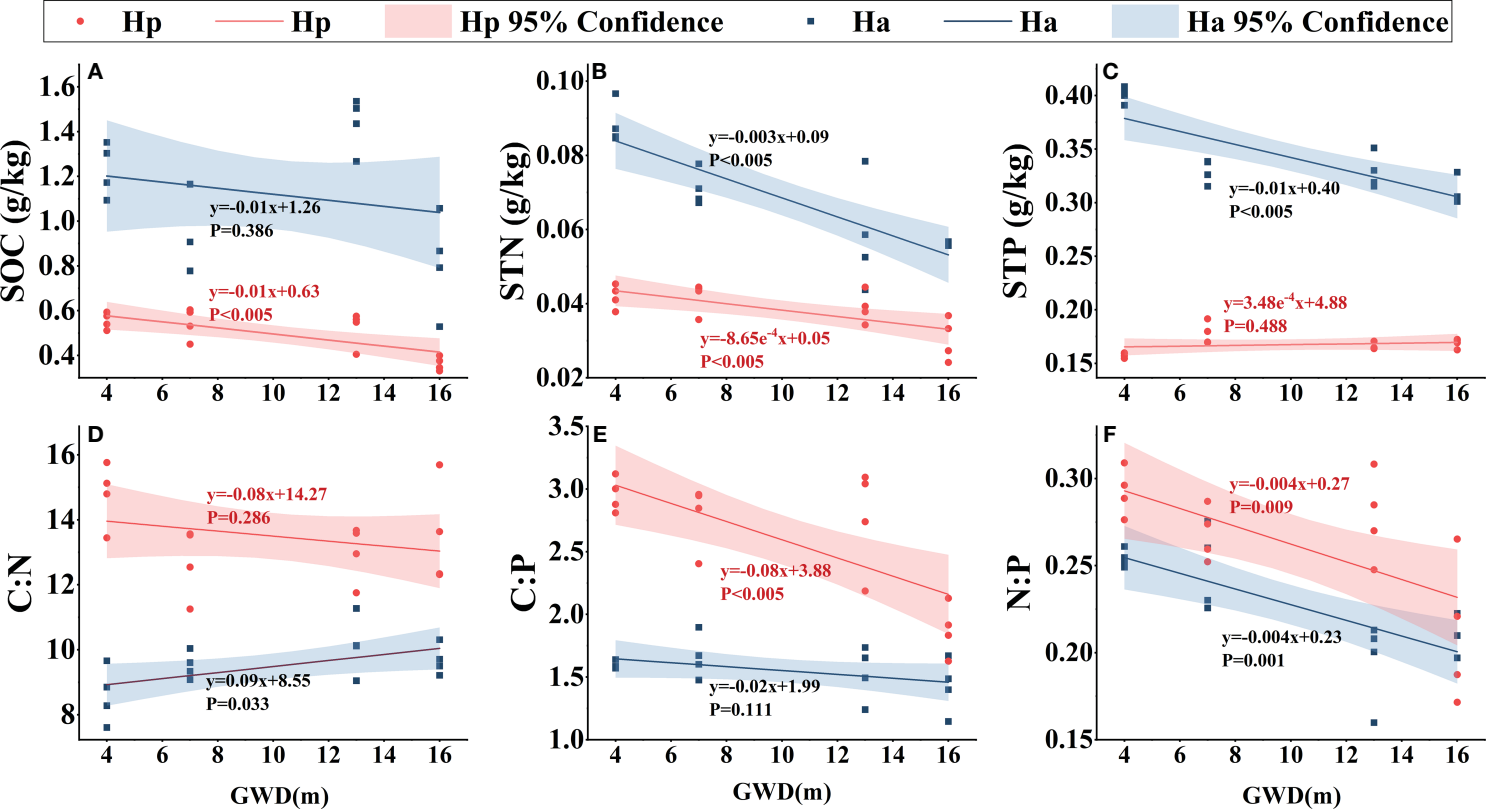
Linear fitting of soil ecological stoichiometry of *H. persicum* and *H. ammodendron* to groundwater depth. Ha, *H. ammodendron*; Hp, *H. persicum*; GWD, groundwater depth. Soil samples from the same site at different depths were averaged and plotted as the sample points. The straight line is the fitted line, and the corresponding formula is the equation of the line. The shaded area is a 95% confidence interval. SOC, soil organic carbon **(A)**; STN, soil total nitrogen **(B)**; STP, soil total phosphorus **(C)**; C:N, soil carbon-nitrogen ratio **(D)**; C:P, soil carbon-phosphorus ratio **(E)**; N:P, soil nitrogen-phosphorus ratio **(F)**.

Except for *H. persicum* assimilated branches LTN, the nutrient contents (LOC, LTN, and LTP) of assimilated branches increased with deeper groundwater (**Figure 5**). Furthermore, assimilated branches stoichiometric ratios varied with groundwater depth. The C:N ratio of assimilated branches increased with groundwater, with a greater increase in *H. persicum* than in *H. ammodendron*, and changed more rapidly. The C:P ratio of assimilated branches remained relatively stable, while the N:P ratio showed a decreasing trend in both species. The slopes of the linear relationships for *H. ammodendron* and *H. persicum* LTP and C:N showed significant differences, while those of LOC and N:P did not, indicating that they changed at similar rates.

**Figure 6:**
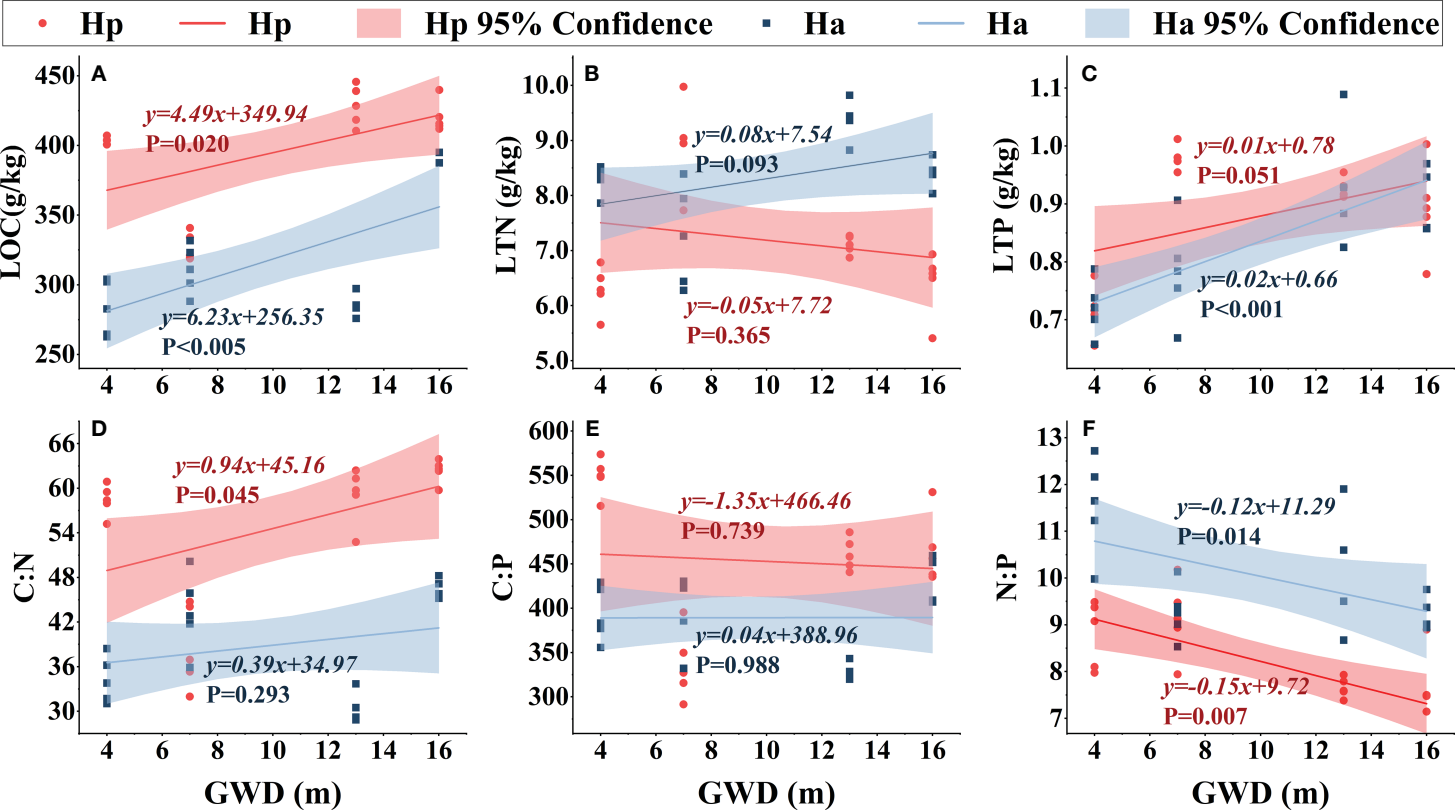
Linear fitting of assimilated branches ecological stoichiometry to groundwater depth. Ha, *H. ammodendron*; Hp, *H. persicum*; GWD, groundwater depth. The straight line is the fitted line, and the corresponding formula is the equation of the fitted line. The shaded area is a 95% confidence interval. LOC, assimilated branches organic carbon **(A)**; LTN, assimilated branches total nitrogen **(B)**; LTP, assimilated branches total phosphorus **(C)**; C:N, assimilated branches carbon-nitrogen ratio **(D)**; N:P, assimilated branches nitrogen-phosphorus ratio **(E)**; C:P, assimilated branches carbon-phosphorus ratio **(F)**.

As groundwater depth increased, SOC, STN, and STP in the soils of *H. persicum* and *H. ammodendron* decreased significantly **Figure 6**. While the slope of the linear fit for *H. ammodendron* SOC was mostly similar to that of *H. persicum*, the change rates of STN and STP were greater. Soil C:N did not vary significantly with groundwater depth. The C:P of soil under the *H. persicum* canopy was significantly lower than that under the *H. ammodendron* canopy, although there was less variation within the soil under *H. ammodendron*. The rate of decrease in N:P of the soil under both species was similar with increasing groundwater depth.

### 3.5 Relationships between assimilated branches and soil ecological stoichiometry

**Figure 7:**
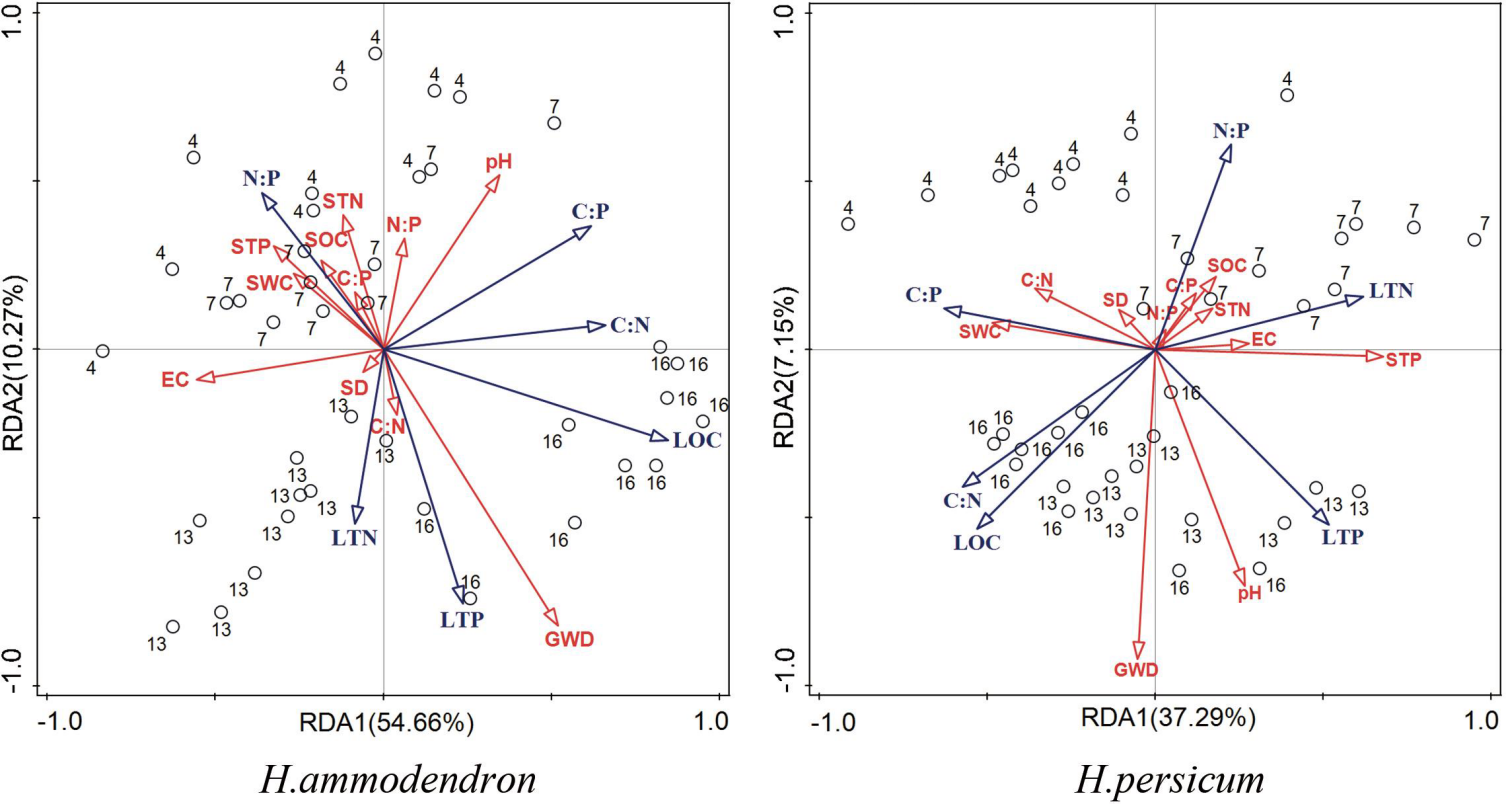
RDA analysis of leaf ecological stoichiometry with groundwater and soil factors. Blue and red arrows indicate leaf and soil ecological stoichiometry, respectively. The circle represents the sample site and the number outside the circle is the depth of groundwater at the sample site.

As shown in **Figure 7**, the RDA ranked the assimilated branches stoichiometry characteristics and soil factors separately for the two plant species. The bubble diagram revealed clear differentiation of assimilated branches characteristics across varying groundwater gradients. *H. persicum* accounted for 37.29% and 7.15% in Axis I and Axis II, respectively. The cumulative amount of plant assimilated branches characteristics explained in the first two axes was 44.44%. For *H. ammodendron*, the corresponding values were 54.66%, 10.27%, and 64.93%. The first two RDA axes effectively reflected most of the relationships between plant assimilated branches ecological stoichiometric characteristics and soil factors, primarily determined by Axis I.

Groundwater depth was found to be the most significant influencing factor, with SWC, pH and STP being ranked highest for their roles in the variation in assimilated branches stoichiometry in both *H. persicum* and *H. ammodendron*. In both species, LOC, LTN, and LTP were positively correlated with groundwater depth. Among the soil factors, *H. persicum* showed a significant positive correlation between soil pH and LTP, as well as between STP and LTN and LTP, while a significant negative correlation between STP and assimilated branches C:P was observed. In *H. ammodendron*, soil pH was found to be significantly negatively correlated with LTN and LTP. The relationship observed between STP and assimilated branches traits was opposite in *H. persicum* compared to *H. ammodendron*. Further significant correlations were also observed between assimilated branches traits such as LTP and N:P, LTN and N:P, and LTN and C:N.

## 4 Discussion

### 4.1 Assimilated branches and soil ecological stoichiometry characteristics

*H. persicum* and *H. ammodendron* LOC, LTN and LTP are lower than the global average for shrubs (461.16 g/kg, 20.1 g/kg, 1.77 g/kg) (He et al., 2019). Assimilated branches C:N and C:P were much higher than average (23.8 and 300.9) (He et al., 2019). Soil SOC, STN, and STP were enormously lower than the global terrestrial average (11.12g/kg, 1.06g/kg, 0.65 g/kg) (Luo et al., 2017). Soil N:P (0.25 ± 0.03, 0.22 ± 0.02) and C:P (2.59 ± 1.90, 1.89 ± 0.11) were much lower than the average in China (2.15, 25.77) (Li et al., 2014). The soil C:N (17.24 ± 2.60) under *H. persicum* was higher than the Chinese average (12.01), while the soil C:N (10.12 ± 0.94) under *H. ammodendron* was lower than the Chinese average (Luo et al., 2017).

C, N and P are important elements for plant growth, development and genetics (Huang et al., 2021). Leaf C:N and C:P can be used as a measure of the plant’s ability to sequester carbon as it absorbs N and P nutrients (Tian et al., 2021). Attributed to lower rainfall and slower nutrient absorption, there was less nutrient storage in the study area, resulting in reduced nutrient accumulation in the assimilated branches. In barren habitats, plants have a conservative survival strategy of maintaining high C:N and C:P ratios to increase nutrient use efficiency to maximize productivity (Zhang et al., 2020).

Assimilated branches N:P can be used as an indicator of vegetation function and nutrient limitation (Tian et al., 2021). Compared to the global mean (He et al., 2019), the assimilated branches N:P of the two *Haloxylon* species was low, which may be the result of low LTN content. According to Gusewell’s 10/20 theory, both *H. ammodendron* and *H. persicum* assimilated branches N:P ratios are mostly less than 10 and show N limitation (Gusewell, 2004). Nevertheless, the supply of plant N and P was obviously insufficient. Plants also have the ability to maintain stable relationships between their intrinsic elemental content and ecological stoichiometry when faced with dramatic changes in environmental conditions (Elser et al., 2010). Therefore, the nutrient limitation of plants should be considered in a wider range of environmental factors rather than being measured by N:P alone.

The element content of soils and their stoichiometric ratios are important indicators of soil composition and quality (Tian et al., 2009). Soil carbon and nitrogen are mainly derived from the decomposition of vegetation litter (Luo et al., 2017). The foliage of *H. persicum* and *H. ammodendron* is degraded, and litter production is much lower than the national average (An and Li, 2014). Soil microbial diversity and biomass are also significantly reduced in arid habitats, and nutrient turnover is slower (Liu et al., 2022). Plants living in barren habitats always have a higher nutrient acquisition cost, so there is often a resorption of nutrients in senescing withered parts, which implies the adaptability of desert plants (Veneklaas et al., 2012). Soil nitrogen can also be derived from rainwater leaching and deposition of atmospheric nitrogen. However, the low precipitation (less than 200 mm per year) in the study area and the longer distance from cities or factories limit soil nitrogen replenishment through this route (Zhou et al., 2012). A combination of many factors results in the reduction of carbon and nitrogen input to the soil. STP is mainly derived from rock weathering (Tian et al., 2021), and the low rate of rock weathering due to drought and low rainfall in the study area contributes to the low soil STP content.

### 4.2 Relationship between the soil ecological stoichiometry and groundwater depth

The nutrient content of soil is mainly affected by litter input, while the physical properties are mainly affected by topography and groundwater supply (Zhou et al., 2012; Chen et al., 2021). As groundwater conditions deteriorate, desert plants adopt stricter defense strategies and produce fewer nutrients in the litter (An and Li, 2014). Increased LOC lead to stimulation of soil organic matter turnover and more carbon input (Li et al., 2018), stimulating microbial activity and soil organic matter decomposition, ultimately resulting in reduced carbon stability; thus, the SOC decreases (Chen et al., 2021). The leaf C:N increasing, so relative N content of litter decreases and causes decomposition to consume soil N (Enriquez et al., 1993). Consequently, the STN content of soil decreases, N limitation increases and leaf N:P decreases significantly. This is also consistent with the results of the RDA: GWD is significantly negatively correlated with soil STN and branches N:P. As structural components of organic matter, C and N have relatively fixed ratios in the process of accumulation and consumption (Huang et al., 2021). As a result, soil C:N did not vary significantly among different groundwater depths and soil layers.

It may be the topography that causes the soil properties to differ between the two plants. Topography is important in controlling hydrological and soil processes because it affects the moisture as well as the accumulation and export of nutrients (Sebastia, 2004). Different topographic leads to the redistribution of soil resources, such as the leaching brings nutrients from the top of the slope to the bottom (Li et al., 2010). Regional groundwater was characterized by high salinity of Cl and SO_4_Cl, leading to the accumulation of salts in the soil due to the effect of evaporative concentration (Pan et al., 2020). So, the SOC, STN and EC under *H. ammodendron* were significantly higher than those under *H. persicum*.

Moreover, other biological processes also affect soil chemical element content. *H. ammodendron* mainly absorbs NO^3-^ from the soil and releases OH^-^, leading to an enhanced alkalinity and greater pH of the soil under the canopy. (James et al., 2005). The salt-rich litter is imported and accumulates in the soil under the canopy, resulting in a salt island effect. In addition, *H. ammodendron* absorbs excess salt ions from the soil to maintain osmotic pressure for survival (Xi et al., 2004). Changes in microbial abundance and metabolic pathways in soil lead to lower turnover rates, then increase accumulation of carbon and nitrogen (Liu et al., 2022). The low C:N under the *H. ammodendron* canopy allows for faster release of N and facilitates uptake (An and Li, 2014), which may explain why both STN and LTN of *H. ammodendron* are significantly greater than those of *H. persicum*.

### 4.3 Adaptations of plants in different groundwater depth

The results revealed a correlation between assimilated branches ecological stoichiometry. A positive correlation was found between LTN and LTP, which is a general pattern in terrestrial plants reflecting the trade-off among leaf attributes (Rong et al., 2015). LTN and LTP were found to be negatively correlated with leaf C:N and C:P, respectively. These reflect the availability of nitrogen and phosphorus nutrients in the assimilated branches of plants, which determine their survival strategy and growth rate (Poorter and Bongers, 2006). Plants employ a trade-off strategy in their assimilated branches ecological stoichiometry by allocating resources and utilizing them efficiently, which ultimately enhances their adaptation to their respective habitats (Harris, 2003).

The density of plants decreased significantly when the depth increased, which proved that their living conditions deteriorated sharply. We speculate that the different sources of water *H. ammodendron* and *H. persicum* at different groundwater depths lead to different degrees of stress, thus showing differences in ecological stoichiometry. The *H. ammodendron* is a groundwater-dependent plant (Dai et al., 2014). However, for *H. persicum*, the survival cost of obtaining groundwater is too high, mainly using deep soil water (Wu et al., 2019a). When the groundwater is shallow depth, the roots can directly absorb groundwater, and a large amount of deep soil water is used in the early part of the growing season (Wu et al., 2019a). Various water sources weaken its stress, so C:N is lower in shallow groundwater (Zhang et al., 2020). Due to the increase of depth, the utilization of groundwater by *H. ammodendron* gradually decreased (Wu et al., 2019a). Precipitation decreased significantly from the edge to the center of the desert, too (Chen, 2017). Soil moisture was reduced by dual effect, drought stress became more serious. Plants adapt to deteriorating water conditions by increasing assimilated branches C:N and nutrient reabsorption (Zhang et al., 2020). Compared with the *H. persicum*, the C:N of *H. ammodendron*, has a more pronounced increase. The *H. persicum* adjust its root distribution to obtain more shallow soil water for more restricted resources, and adapt to the increase in groundwater depth (Xu et al., 2017). In this region, not only is the soil N supply limited, but the P content is also sparse. Under low P supply conditions, plants preferentially allocate P to photosynthetic cells to ensure photosynthetic efficiency for survival (Ding et al., 2021). Under intense drought, plants tend to accumulate more nutrients in branches. With the accumulation of P more than N, the N:P is lower in deep groundwater (Cordell et al., 2001). This storage process leads to a decrease in the assimilated branches N:P ratio. The increase of LOC, C:N and C:P demonstrates that plants have adopted stronger defense strategies to survive in an increasingly drought-stressed environment (Poorter and Bongers, 2006; Rong et al., 2015).

Therefore, we believe that the two *Haloxylon* adapt to changes in groundwater depths through the allocation of water use and branches ecological stoichiometry.

## 5 Conclusions

Both *Haloxylon persicum* and *Haloxylon ammodendron* in the region are limited by N and P. The branches and soil C, N, and P contents were lower than the national averages in China, while the C:N and C:P ratios were significantly higher. Clear trends were observed in the ecological stoichiometric characteristics of the assimilated branches and soil with varying groundwater depths. The LOC, LTN, and LTP contents increased, while the ratios differed: C:N increased, C:P remained stable, and N:P decreased. The soil characteristics showed almost downward trends. The topography and other biological processes cause the green branches traits and soil properties differ between the two plants to some extent. The depth of groundwater was found to be a significant environmental factor affecting assimilated branches ecological stoichiometry. A significant correlation was observed between assimilated branches ecological stoichiometry and soil properties. SWC, pH, and STP were the main common influencing factors for both studied species, indicating that soil physicochemical properties have a significant influence on the accumulation of elements in assimilated branches. The two *Haloxylon* adapt to changes in groundwater depths through the allocation of branches ecological stoichiometry. Moreover, the branches improved their adaptation to soil water and salt, as they employed competition and defense strategies through mutual trade-offs.

The results of this study reveal the response mechanisms of *Haloxylon persicum* and *Haloxylon ammodendron* to changes in groundwater depth to some extent, contributing to a better understanding of their adaptation strategies in arid environments. This study can provide scientific evidence for ecological conservation, restoration, and management processes in desert areas under changes in local hydrological conditions in the future.

## Data availability statement

The original contributions presented in the study are included in the article/**Supplementary Material**. Further inquiries can be directed to the corresponding author.

## Author contributions

All authors contributed to the study conception and design. The idea was provided by YL. Material preparation and data collection was conducted by XW, under the guidance of YL. Data analysis and drawing of the graphics were performed by XYW, YG and PW. The first draft of the manuscript was written by XYW and directed and revised by XW. All authors commented on previous versions of the manuscript and approved the final manuscript.
